# Hyperthermic Intraperitoneal Chemotherapy with Melphalan: A Summary of Clinical and Pharmacological Data in 34 Patients

**DOI:** 10.1155/2012/827534

**Published:** 2012-07-03

**Authors:** Lana Bijelic, Paul H. Sugarbaker, O. Anthony Stuart

**Affiliations:** ^1^Washington Cancer Institute, Washington, DC 20010, USA; ^2^MedStar Health Research Institute, Washington, DC 20010, USA

## Abstract

Cytoreductive surgery (CRS) with hyperthermic intraperitoneal chemotherapy (HIPEC) is a treatment option for peritoneal metastases. The optimal agents for HIPEC have not been established. Melphalan is a drug with broad activity and a favorable profile for intraperitoneal application. The purpose of this study is to review our experience using melphalan for HIPEC. Pharmacologic data was obtained. Thirty four patients who underwent CRS for peritoneal metastases received melphalan for HIPEC between 2003 and 2011. The first 10 patients received 70 mg/m^2^; subsequent 24 received 60 or 70 mg/m^2^. The mean PCI was 21 ± 7. Twenty-eight patients (83%) had a CC score of 1 or 2. The mean length of stay was 18 ± 2 days. Nine patients (26%) had a grade 3 and 6 (17%) had grade 4 morbidity. There were no postoperative deaths. The pharmacologic analysis of plasma to peritoneal fluid levels of melphalan showed an AUC ratio of 33 while the tumor nodules to peritoneal ratio was 8. Melphalan is an acceptable agent for use in HIPEC. The morbidity of intraperitoneal melphalan at the dose of 60–70 mg/m^2^ appears acceptable. Further studies comparing the effectiveness of melphalan and other HIPEC agents are needed.

## 1. Introduction

Cytoreductivesurgery, combined with heated intraperitoneal chemotherapy (HIPEC), is a treatment modality that can provide long-term survival for selected patients with peritoneal metastases from gastrointestinal cancer and mesothelioma [1–3]. The best outcomes are achieved in patients who have a complete surgical removal of the peritoneal deposits [4, 5]. Unfortunately, recurrence on the peritoneal surface is common even after successful complete cytoreduction [6–8]. Therefore, efforts should be directed to improve the effectiveness of intraperitoneal chemotherapy as a way to maintain the disease control obtained with complete cytoreduction. 

While HIPEC is almost universally used as an integral component of the cytoreductive procedure, there is little consensus on the optimal regimen. There is variability in both the chemotherapy agents and doses used among treatment centers due to a lack of studies directly comparing different HIPEC regimens. Treatment centers often base their choice of HIPEC regimen on theoretical principals and pharmacologic data.

Melphalan is an antineoplastic alkylating agent that causes the formation of interstrand DNA crosslinks and shows a marked increase in activity with heat [9, 10]. For this reason, it remains the principal agent used in isolated limb perfusion for the treatment of in-transit metastases of melanoma [11]. We have previously shown in an animal model that the intraperitoneal administration of melphalan combined with heat is effective in delaying tumor growth and that the effect of hyperthermia on the pharmacokinetics and tissue distribution of intraperitoneally administered melphalan indicated increased intraabdominal tissue concentrations [12]. 

Therefore, we sought to evaluate the feasibility of using melphalan for heated intraperitoneal chemotherapy combined with cytoreductive surgery for the treatment of peritoneal metastases from appendix and colorectal cancer as well as mesothelioma.

## 2. Methods

 All patients, undergoing cytoreductive surgery with HIPEC utilizing melphalan, were identified by searching our prospectively maintained database. From July 2003 until July 2011, 34 patients received HIPEC with melphalan. The first 10 patients were treated as part of a prospective, single-institution phase I trial approved by the Institutional Review Board. 

Cytoreductive surgery was performed by the senior author in all cases and consisted of peritonectomies and visceral resections performed as needed to achieve complete tumor removal whenever possible as previously described [13]. After all resections were completed, the patients underwent HIPEC with melphalan for 60 or 90 minutes. Melphalan was given at a dose of 50–70 mg/m^2^ in 1.5 L/m^2^ of 1.5% dextrose peritoneal dialysis solution. The dose of melphalan was chosen based on the number of cycles of systemic chemotherapy that the patients received prior to cytoreductive surgery and their performance status. HIPEC was performed using the open coliseum method except in select patients with incomplete cytoreduction in whom the closed method was used to provide increased intraabdominal pressure [14]. One inflow catheter and four outflow catheters were used to circulate the chemotherapy solution in both the open and closed method. The temperature of the chemotherapy solution was maintained at 41-42°C inside the abdomen and continuously monitored with two temperature probes. 

Early postoperative intraperitoneal chemotherapy with 5-FU was used in four patients who did not receive any prior systemic or intraperitoneal chemotherapy during the phase I trial. Following completion of the phase I trial, HIPEC with melphalan was used in patients with recurrent disease being treated with repeat cytoreductive surgery and HIPEC. This group of patients did not receive EPIC. Perioperative variables, including the peritoneal cancer index (PCI), extent of cytoreductive surgery, completeness of cytoreduction (CC score) and a detailed assessment of morbidity by grade, and organ system for each patient, were prospectively assessed and entered into a database. 

Pharmacological assessments were done on the first 10 patients enrolled in the phase I trial and an additional 10 patients treated afterwards. In patients who underwent pharmacologic analysis, samples of peritoneal fluid, blood, urine, and, where available, tumor nodules were collected immediately prior and every 15 minutes during HIPEC. Melphalan concentration was assessed using high-performance liquid chromatography (HPLC) within 24 hours of collection. Melphalan concentrations were determined using a modification of the HPLC method described by Norda et al. [15] We used a Shimadzu LC7A instrument equipped with a SPD-6AV (UV-VIS) detector set at 270 nm along with a C-R6a Chromatopac data processor. A Dynamax reversed-phase C_18_ column (150 × 4.6 mm^2^) of Microsorb 100° 5 *μ*m particles was used coupled to a guard column of the same chemical consistency (Varian Associates, Walnut Creek, CA, USA). The mobile phase consisted of an isocratic mixture of 30% acetonitrile in 0.005 M NaH_2_PO_4_ with the pH adjusted to 3.5 with phosphoric acid. The flow rate was set at 1.2 mL/min and the volume of sample injections was 50 *μ*L. All solvents used were HPLC grade (Fisher Scientific, Norcross, GA, USA).

## 3. Results

Thirty-four patients received heated intraoperative intraperitoneal melphalan between July 2003 and July 2011. There were 20 females and 14 males. Twenty-three patients had appendiceal carcinoma, 6 had mesothelioma, 2 colon cancer, 2 ovarian cancer, and 1 had urachal carcinoma. Eleven patients received melphalan at their first cytoreduction with HIPEC while 23 had repeat CRS+HIPEC for recurrent disease. 

The mean PCI was 28 for patients who received melphalan at the time of their first cytoreductive procedure and 18 for patients who had repeat cytoreduction.

Twenty-one patients had a CC score of 1, seven had a CC score of 2, and six had a CC score of 3. All the demographic and perioperative data is summarized in Table 1. 

**Figure 1:**
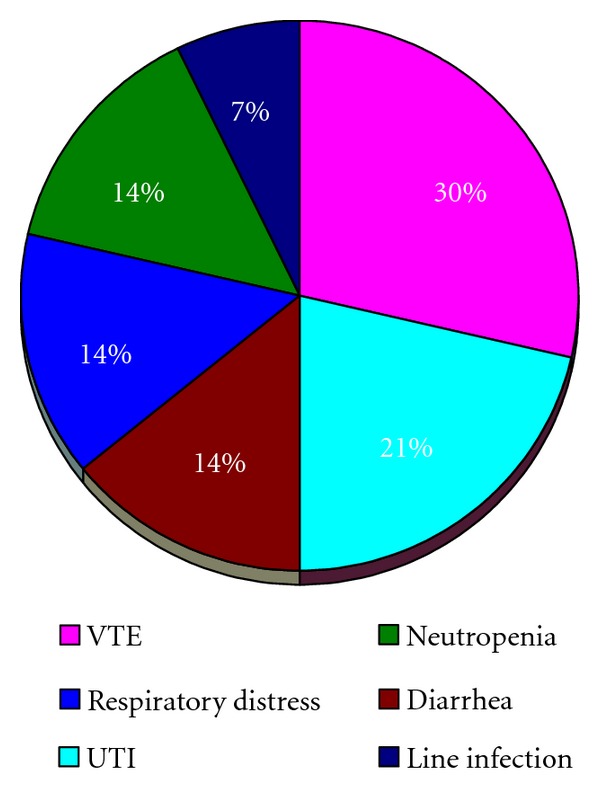
Grade 3 complications observed during the postoperative period in 34 patients treated with cytoreductive surgery and HIPEC with melphalan.

**Figure 2:**
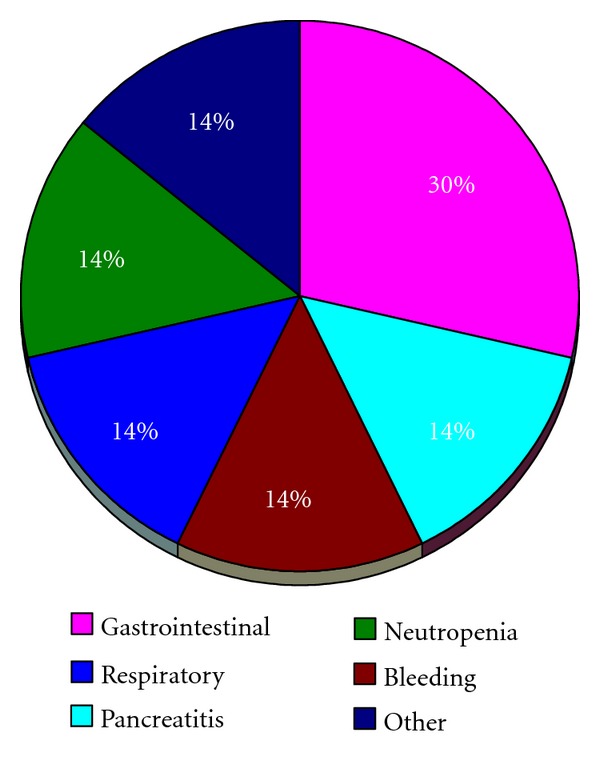
Grade 4 complications observed during the postoperative period in 34 patients treated with cytoreductive surgery and HIPEC with melphalan.

The number of peritonectomies performed ranged from 0 to 4 (mean 1.05). The number of visceral resections performed ranged from 0 to 6 (mean 2.26). The mean length of stay was 18 ± 2 days. Nine patients (26%) had a grade 3 complication in the postoperative period. The following grade 3 complications were observed: deep vein thrombosis in 4 instances, urinary tract infection in 3, diarrhea in 2, respiratory distress in 2, neutropenia in 2, and catheter associated bloodstream infection in 1 (Figure 1). Six patients (17%) had grade 4 morbidity: there was 1 fistula and 1 Hartmann's pouch leak, 1 severe pancreatitis, 1 occurrence of postoperative bleeding, 1 case of ARDS, 1 lower extremity compartment syndrome, and 1 grade IV neutropenia (Figure 2). There were no postoperative deaths. On univariate analysis, the dose of melphalan, the number of visceral resection, and the number of peritonectomies were associated with an increased incidence of grade 4 complications, while the peritoneal cancer index and the use of EPIC were not (Table 2).

**Figure 3:**
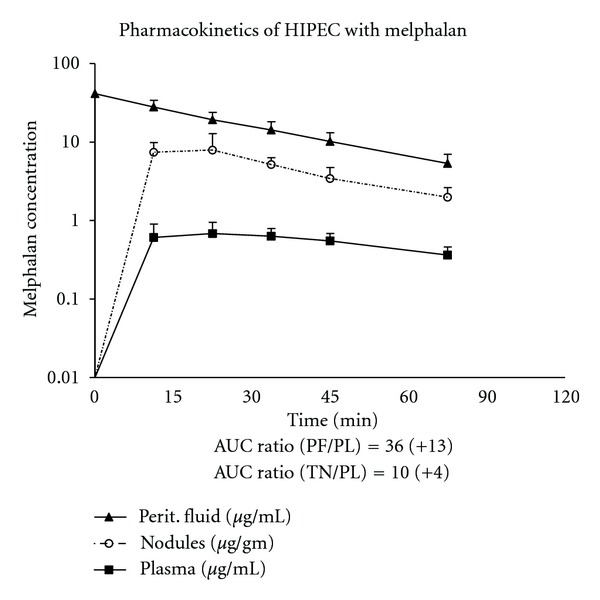
Pharmacologic analysis showing peritoneal fluid, plasma, and tumor nodule levels of melphalan in 20 patients treated with cytoreductive surgery and HIPEC with melphalan.

The mean total dose of melphalan received was 116 ± 21 mg. Five patients were treated with 50 mg/m^2^, 17 patients received 60 mg/m^2^, and 10 received 70 mg/m^2^ (the dose/m^2^ data was unknown for 2 patients). The pharmacologic analysis was carried out in 20 patients. During 90 minutes of HIPEC, an average of 85.7 ± 5.2% of melphalan was absorbed. The average absorption at 60 minutes of treatment was 75.2 ± 7.5%. The average peritoneal fluid AUC over 90 minutes of HIPEC was 1541 ± 295 *μ*g/mL while the average plasma AUC was 46 ± 13. The average peritoneal fluid to plasma AUC ratio was 35 ± 13 (Figure 3). 

**Figure 4:**
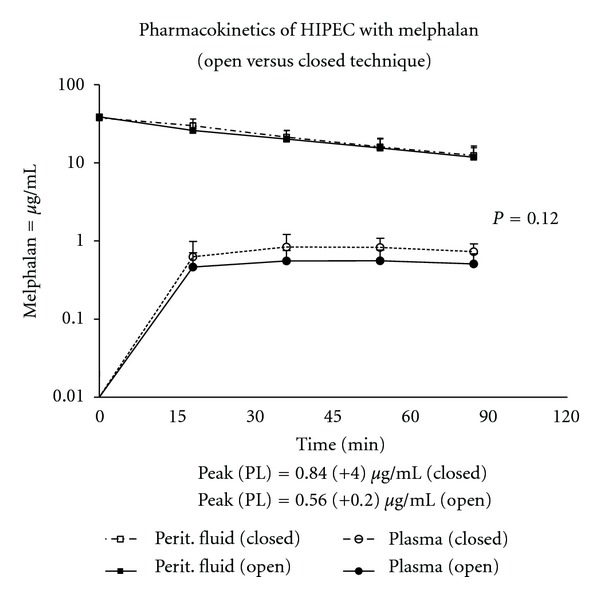
Pharmacokinetics of hyperthermic intraperitoneal melphalan in 3 patients in whom the closed technique was used compared to 12 patients treated with the open technique. The difference in the plasma levels was not statistically significant (*P* = 0.12).

There were three patients who received intraperitoneal melphalan using the closed technique who had a complete pharmacologic evaluation. The pharmacologic data on these patients was compared to 12 patients treated with hyperthermic intraperitoneal melphalan using the open technique. The melphalan levels in these patients are shown in Figure 4. The plasma levels of melphalan were slightly increased in the closed technique as compared to the open; however; these results were not statistically significant.

## 4. Discussion

The rationale for using local regional chemotherapy following cytoreductive surgery for peritoneal metastases is based on the well-documented pharmacokinetic advantage of intraperitoneal delivery that results in high peritoneal fluid levels and comparatively low systemic levels [16]. From a theoretical standpoint, the choice of agents for use in HIPEC should take maximal advantage of this principle. The pharmacokinetics of intraperitoneal melphalan have been studied by Howell at al. under normothermic conditions showing approximately 90% systemic absorption at 4 hours [17]. However, we have previously shown in an animal model that the addition of hyperthermia increases the rate of systemic absorption [12]. In the current study, we performed melphalan HIPEC for 90 minutes in the first 18 patients. An analysis of the pharmacology shows there was approximately 85% absorption at 90 minutes compared to 75% at 60 minutes. Considering, there is only a 10% additional systemic exposure in the last 30 minutes of treatment, we have modified the duration of melphalan HIPEC to 60 minutes. Our pharmacologic analysis also confirms a favorable peritoneal fluid to plasma AUC ratio of 36 when melphalan is used for HIPEC. This AUC ratio compares favorably with other chemotherapy agents that are commonly used for HIPEC, such as mitomycin C with a peritoneal fluid to plasma AUC ratio of 24 and cisplatin with an AUC ratio of 8 [18]. Urano and Ling studied the effect of hyperthermia on the cytotoxicity of melphalan showing maximal thermal enhancement of melphalan at 41.5°C [10]. Therefore, we have used a target temperature of 41-42°C for the perfusate in the current study. 

Previous studies of normothermic intraperitoneal melphalan by Howell at al. showed the maximal tolerated dose to be approximately 70 mg/m^2^ [17]. Our early experience with melphalan HIPEC at a dose of 70 mg/m^2^ seemed to suggest an increased incidence of perioperative morbidity. Based on this clinical observation, we empirically decreased the dose to 60 mg/m^2^. The current study provides an analysis of all the patients we have treated with heated intraperitoneal melphalan. The univariate analysis of clinical variables associated with grade 4 morbidity confirms our clinical impression and shows a statistically significant increase in grade 4 morbidity for patients treated with 70 mg/m^2^ of melphalan. Other factors associated with increased morbidity were the number of peritonectomies and visceral resections. The morbidities observed in this group of patients were very similar to those observed in our recent study of patients undergoing cytoreductive surgery and HIPEC with mitomycin C and doxorubicin [19]. We did not observe morbidities specific to the use of melphalan. 

Heated intraperitoneal chemotherapy can be delivered using the open or the closed abdomen technique. There have been no studies showing a clear advantage of one technique over the other. In our practice, we use both techniques depending on the clinical scenario. The closed technique is typically used when no cytoreduction of small bowel surfaces needs to be done or in patients with incomplete cytoreduction in whom the increased intra-abdominal pressure may provide improved tissue penetration of the chemotherapy solution. In this study, we compared the pharmacology of melphalan used with the open versus the closed abdomen technique. One might suspect that the closed technique would demonstrate an increased clearance of chemotherapy from the abdominal/pelvic space into the plasma. There is an increase in the total diffusion surface because the surface of the anterior abdominal wall and the surface of the skin and subcutaneous tissue are exposed to the chemotherapy in the closed method. These surfaces are only intermittently exposed with the open method. Also, there is a slight increase in pressure within the abdomen with the closed technique. However, pharmacologically, these expected differences were not observed and the pharmacology of melphalan is virtually identical regardless of the HIPEC technique used. We have previously shown that more significant changes in the diffusion surface, such as those seen with large visceral resections or in patients with a contracted peritoneal space do have an impact on the clearance of mitomycin C and doxorubicin from the peritoneal cavity [20, 21].

This study was primarily designed to provide information about the safety of hyperthermic intraperitoneal melphalan and to help establish an optimal dose and duration of melphalan HIPEC. It is not possible to make judgments regarding the clinical efficacy of melphalan in terms of its impact on survival from the current series. The patient population, in this study, is heterogeneous in terms of primary diagnosis as well as whether the cancer was primary or recurrent. Therefore, an analysis of survival, following HIPEC with melphalan, would not provide clinically useful insights into its efficacy. 

In conclusion, our experience in 34 patients treated with hyperthermic intraperitoneal melphalan suggests that melphalan is a reasonable chemotherapy agent to use for HIPEC with a favorable pharmacologic and safety profile. We suggest a dose of 60 mg/m^2^ for 60 minutes. Based on our experience, melphalan should be included in future studies comparing different HIPEC agents, especially for patients with peritoneal recurrence following initial cytoreduction plus HIPEC.

## Figures and Tables

**Table 1:** Demographic and perioperative data on 34 patients treated with cytoreductive surgery and heated intraperitoneal chemotherapy with melphalan.

Gender	
Male	14
Female	20
Age (mean)	46.1
Primary diagnosis	
Appendix cancer	23
Mesothelioma	6
Colon cancer	2
Ovarian cancer	2
Urachal cancer	1
Peritoneal cancer index (PCI)	
Mean	21
Range	4–39
Completeness of cytoreduction score	
CC-1	21
CC-2	7
CC-3	6
Dose of melphalan	
50 mg/m^2^	5
60 mg/m^2^	17
70 mg/m^2^	10

**Table 2:** Univariate analysis of clinical factors associated with grade 4 postoperative morbidity in 34 patients treated with cytoreductive surgery and HIPEC with melphalan.

Clinical variable	Grade 4 morbidity		*P* value
	Yes	No
Dose of melphalan			0.01
50 mg/m^2^	0
60 mg/m^2^	0
70 mg/m^2^	4
Number of peritonectomies			<0.001
≤2	1
>2	5
Number of visceral resections			0.002
≤2	0
>2	6
Peritoneal cancer index			0.38
≤20	1
>20	5
EPIC			0.13
Yes	2
No	26
